# Simultaneous integrated boost with stereotactic radiotherapy for dominant intraprostatic lesion of localized prostate cancer: a dosimetric planning study

**DOI:** 10.1038/s41598-020-71715-2

**Published:** 2020-09-07

**Authors:** Yeon Joo Kim, Kyoung Jun Yoon, Young Seok Kim

**Affiliations:** Department of Radiation Oncology, Asan Medical Center, College of Medicine, University of Ulsan, 88, Olympic-ro 43-gil, Songpa-gu, Seoul, 05505 Republic of Korea

**Keywords:** Prostate cancer, Radiotherapy

## Abstract

Dominant intraprostatic lesion (DIL) has been known as the most common local recurrence site of prostate cancer. We evaluated the feasibility of simultaneous integrated boost (SIB) to DIL with CyberKnife stereotactic body radiotherapy (CK-SBRT). We selected 15 patients with prostate cancer and visible DIL and compared 3 plans for each patient: 1) No boost plan of 35 Gy to prostate, 2) DIL_40 plan of SIB 40 Gy to DIL and 35 Gy to prostate, and 3) DIL_45 plan with 45 Gy to DIL and 35 Gy to the prostate in 5 fractions. All targets satisfied with the prescription coverage per protocol. However, some patients failed to meet the D_max_ of the rectum in DIL_40 plans (n = 4), and DIL_45 plans (n = 6). Violations of bladder constraints occurred in four DIL_45 plans. Consequently, the DIL boost with SBRT was possible in 73% of patients with DIL_40 plans, and 60% of patients with DIL_45 plans without any violation of normal organ constraints. All patients who experienced constraint violations had DILs in posterior segments. DIL boost using CK-SBRT could be an option for localized prostate cancer patients. For patients who had DIL in posterior segments, a moderate dose escalation of 40 Gy seemed appropriate.

## Introduction

It is well known that dose escalation has decreased biochemical and local failure in patients with localized prostate cancer^1,2^. Nevertheless, still about a quarter of patients who underwent definitive external beam radiotherapy (EBRT) experienced recurrence after treatment. A higher dose to the entire prostate is limited by adjacent normal tissue tolerance, and there were studies that the most common local recurrence site of prostate cancer following conventional fractionated EBRT was the dominant intraprostatic lesion (DIL)^3,4^. Therefore, selective dose escalation to DIL could be a promising strategy to increase tumor control with a minimal increment of toxicity. With the concept, several studies reported the result of the RT boost to the DIL. A meta-analysis demonstrated the data from the 11 therapeutic studies that gave an RT boost of ≥ 90 Gy to the DIL in 988 patients^5^. The median 5-year biochemical recurrence-free survival (BCRFS) was 84% (range, 78%-92%), and the gastrointestinal (GI) and genitourinary (GU) toxicities were acceptable.

Based on the radiobiologic studies which demonstrated that α/β ratio of prostate cancer may be as low as 1.5 Gy^6,7^, hypofractionation scheme using larger fraction size has been used for the treatment of prostate cancer. An extreme form of hypofractionation is stereotactic body radiotherapy (SBRT) with a single dose of 7 Gy or higher for 4–5 fractions. Early studies already reported favorable results of SBRT^8–12^. However, there were few studies on simultaneous integrated boost (SIB) to DIL with SBRT^13^. The robotic non-coplanar technique using CyberKnife (CK) and gantry-based techniques such as volumetric modulated arc therapy (VMAT) can be used for SBRT planning. Compared to VMAT, CK has the benefit of intra-fractional movement control. A previous study also demonstrated that CK could achieve the same dose distribution with high dose rate brachytherapy^14^.

The present study was conducted to evaluate the feasibility of SBRT combined with the DIL boost utilizing CK. Also, we tried to find the appropriate dose prescription satisfying both target coverage and organs at risk (OAR) constraints and to demonstrate for which patient violation of normal organ constraints would occur (Table 1).

**Table 1:** Dose prescription and OAR constraints used for the planning.

Type		Parameter	Prescription or constraints
Target volume	DIL	D_95%_	95% of the PD to DIL
	Whole prostate	D_95%_	100% of PD (35 Gy)
	PTV	D_99%_	80% of PD (28 Gy)
OAR	Rectum	D_max_	100% of the PD (35 Gy)
		V_88% of PD (30.8 Gy)_	< 1 cc
	Bladder	D_max_	110% of the PD (38.5 Gy)
		V_102% of the PD (35.7 Gy)_	< 1 cc
	Urethra	D_max_	No boost: 110% of the PD (38.5 Gy)
			DIL_40: 115% of the PD (40.25 Gy)
			DIL_45: 120% of the PD (42 Gy)

*DIL* dominant intraprostatic lesion, *OAR* organ at risk, *PD* prescription dose, *PTV* planning target volume.

## Results

Table 2 showed the characteristics of patients and DIL. The median age of 15 patients was 66 years (range, 56–82), and the median initial PSA was 5.6 ng/mL (3–10.6). T stages were various with T2a (n = 10), T2b (n = 1), and T2c (n = 4) disease. Four patients presented with Gleason score (GS) 6, 10 patients had GS 7, and one patient diagnosed with GS 9. Four patients with T2c disease had 2 DILs, and one patient had a single tumor involving two segments. The locations of DIL were various, and more than half were posterior segments (12 out of 20 locations). Median DIL volume was 1.3 cc (range, 0.7–6.6), and median prostate volume was 46.2 cc (range, 18.9–63.4 cc).

**Table 2:** Characteristics of patients and DILs.

Characteristics		Value
Age (years)	Median (range)	66 (56–82)
PSA (ng/mL)	Median (range)	5.6 (3–10.6)
T stage	2a	10
	2b	1
	2c	4
Gleason score	6	4
	7	10
	9	1
Location of DIL^b^	2a^a^	4
	3a	1
	3p^a^	3
	5a	1
	5p	2
	6a	2
	6p	2
	8p	3
	9p	1
	10p	1
DIL volume (cc)	Median (range)	1.3 (0.7–6.6)
Prostate volume (cc)	Median (range)	46.2 (18.9–63.4)
DIL / prostate volume ratio	Median (range)	0.03 (0.01–1.21)
PTV volume (cc)	Median (range)	70.0 (28.8–88.0)

*DIL* dominant intraprostatic lesion, *PSA* prostate specific antigen.

^a^One patient had a single tumor involving both 2a and 3p segments.

^b^Four patients had two lesions in separate segments.

**Figure 1:**
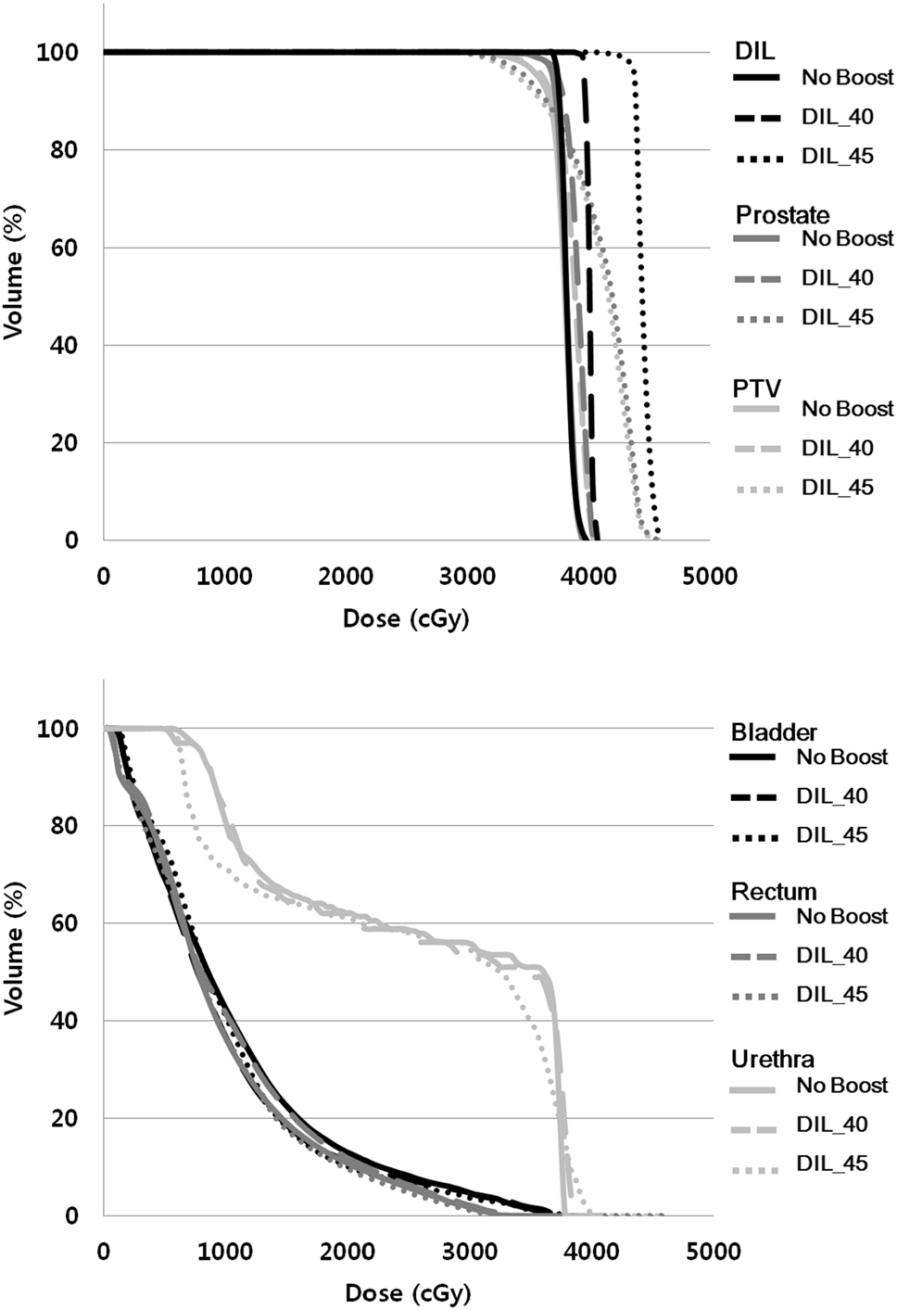
(1) Dose-volume histogram (DVH) of targets and (2) DVH of Organs at risk in a 71-year old man with T2a prostate cancer located in 2a segment.

The results of the planning study were shown in Table 3. All target volumes (DIL, prostate, and planning target volume [PTV]) could achieve the planning goal as we expected. The maximum dose points were all located in DIL. Figure 1 showed the dose-volume histogram of one of the patients. Dose to DIL was escalated (Fig. 1.1), while irradiation to bladder, rectum, and urethra remained under constraints (Fig. 1.2). There were also cases with violations in OAR constraints of DIL_40 and DIL_45 plans. In the aspect of D_max_ of the rectum, 4 patients exceeded 35 Gy in DIL_40 plans (range, 36.4–38.4 Gy), and 2 more patients failed to meet constraints in DIL_45 plans (range, 37.7–43.2 Gy). Similarly, there were patients who demonstrated more than 1 cc of V_30.8 Gy_ of rectal volume in DIL_40 plans (n = 3; range, 1.69–5.09 cc) and DIL_45 plans (n = 5; range, 3.2–7.4 cc). Patients who resulted in rectal constraint violation were all had DIL in posterior segments.

**Table 3:** Dosimetric comparison.

Structure	Parameter	Plan (mean ± SD)			Overall	Multiple comparison		
						1 vs. 2	1 vs. 3	2 vs. 3
		1. No boost	2. DIL_40	3. DIL_45	*P*-value	*P*-value		
DIL	D_95%_ (Gy)	35.67 ± 1.49	38.85 ± 0.80	42.89 ± 1.09	< 0.001	0.001	0.001	0.001
Prostate	D_95%_ (Gy)	35.09 ± 0.60	35.10 ± 0.81	35.23 ± 0.68	0.725	0.443	0.778	0.887
PTV	D_99%_ (Gy)	31.10 ± 0.81	30.95 ± 1.36	29.88 ± 1.43	0.008	0.842	0.036	0.009
	D_max_ (Gy)	39.45 ± 0.22	41.94 ± 2.44	45.77 ± 1.50	< 0.001	0.001	0.001	0.002
	CO	91.18 ± 2.28	89.46 ± 3.69	88.67 ± 4.13	0.109	0.115	0.081	0.393
	CI	1.20 ± 0.04	1.20 ± 0.07	1.35 ± 0.12	< 0.001	0.483	0.001	0.001
Rectum	D_max_ (Gy)	33.28 ± 0.39	34.96 ± 1.48	37.15 ± 3.44	< 0.001	0.001	0.001	0.003
	D_30.8 Gy_	0.70 ± 0.20	1.00 ± 1.23	2.14 ± 2.67	0.127	0.02	0.036	0.256
Bladder	D_max_ (Gy)	36.46 ± 1.05	37.31 ± 0.83	39.13 ± 2.07	< 0.001	0.006	0.001	0.013
	D_35.7 Gy_ (cc)	0.41 ± 0.30	0.46 ± 0.30	1.17 ± 1.50	0.155	0.691	0.023	0.053
Urethra	D_max_ (Gy)	37.78 ± 0.17	39.27 ± 0.91	41.8 ± 2.16	< 0.001	< 0.001	< 0.001	< 0.001
Number of nodes		358 (291–387)	377 (291–416)	316 (190–456)	0.063	0.242	0.122	0.042
Treatment time per fraction (minutes)		71 (65–73)	72 (65–75)	70 (51–79)	0.191	0.214	0.313	0.122

*CI* conformity index, *CO* tumor coverage, *DIL* dominant intraprostatic lesion, *PTV* planning target volume.

Violations of bladder and urethra constraints only occurred in DIL_45 plans: 4 cases of exceeding D_max_ of 38.5 Gy (range, 39.3–41.9 Gy), 2 cases of more than 1 cc of V_35.7 Gy_ (range, 2.1–6.3 cc), and 5 patients of maximal urethra dose over 42 Gy (range, 43.2–45.5 Gy). Likewise, the DIL of the patients was located in posterior segments. Consequently, the DIL boost with SBRT was possible in 73% of patients with DIL_40 plans, and 60% of patients with DIL_45 plans without any violation of normal organ constraints.

## Discussion

The present study compared three CK-SBRT plans in each patient with information on the locations, volumes, and numbers of the DIL. All patients who resulted in OAR constraint violations had posterior segment DIL. Using DIL_40 plans, 3 out of 4 cases for rectal D_max_ violation showed the range of violations lower than 2 Gy. Two out of 3 cases for V_30.8 Gy_ > 1 cc were also exceeding range within 2 cc. We had to point out that the other study groups used more generous OAR constraint than the present study; rectal D_max_ ranged from 36 to 38 Gy (The present study, 35 Gy), rectal V_32.3_–_36 Gy_ < 1 cc (The present study, V_30.8 Gy_ < 1 cc), and bladder D_max_ 41.8 Gy (The present study, 38.5 Gy), V_38 Gy_ < 1 cc or V_37 Gy_ < 10 cc (The present study, V_35.7 Gy_ < 1 cc)^15–17^. Kim et al. reported the dosimetric predictors of rectal tolerance observed in a phase 1–2 trial of dose-escalated SBRT with 45, 47.5, and 50 Gy in 5 fractions to the whole prostate^18^. Grade ≥ 3 late rectal toxicity was related to V_50 Gy_ > 3 cc, and > 35% circumference of rectal wall to 39 Gy. Grade ≥ 2 acute rectal complication was correlated with > 50% circumference to 24 Gy. In our study, dose irradiated to 50% of the rectal volume was below 18.5 Gy. For the DIL boost, we are going to establish revised OAR constraints with 2 Gy-relaxation for rectal D_max,_ and V_30.8 Gy_ of rectum < 2 cc, which able 93% of the patients to be treated with DIL_40 plan. Since 70% of the prostate cancers arise in posterior/peripheral zone^19^, for the further dose escalation with DIL_45 plans, we consider methods for rectal sparing such as the use of spacer^20^.

The number and volume of DIL were not significant factors, and the location alone mattered. Four patients had two DILs. Two patients who one or more DIL(s) was/were located in anterior segments met all OAR constraints for all plans. On the other hand, the rest failed to satisfy one or more constraints. Volumes of the DIL were mostly below 3 cc in the present study, likewise in previous Aluwini et al. (mean DIL volume 1.2 cc, range 0.46–4.1)^13^. Only two patients had the DIL volumes of 3.7 and 6.6 cc, respectively, and all plans were satisfied with the prescription coverage and OAR constraints.

Similar to our study, Tree et al. compared the two methods of SBRT delivery, CK and VMAT. The dose scheme was 47.5 Gy to DIL while maintaining 36.25 Gy to the whole prostate in 5 fractions^15^. Both CK and VMAT planning produced clinically acceptable plans if the same PTV margins were applied. However, in case further margin for intra-fraction motion control was applied to the VMAT system, more violations of OAR constraints were observed in VMAT planning. They provided D50, D20, D10, D5 and hottest 1 cc of the rectum and the measures were comparable to those of our plans^15^.

We summarized the clinical outcomes of SBRT for prostate cancer in Table 4.8–13 Aluwini et al.^13^ alone reported data on DIL boost in SBRT setting with a dose regimen of 38 Gy delivered to prostate and 44 Gy delivered to DIL in 4 fractions. With a median follow-up of 23 months, BCRFS was 100%, and ≥ grade 2 late GI toxicity was 3% and ≥ grade 2 late GU toxicity was 16%. Although 2-year outcomes were feasible, long-term outcomes are wanting. Also, we should be aware of a higher grade 3 GU toxicity rate of 6% compared to those of the other studies except for Hannan et al.^11^ (0–2.4%). This might attribute to a higher dose per fraction (9.5 Gy) compared to the other series (mostly below 8 Gy per fraction). Hannan et al.^11^ reported the results of dose-escalated SBRT (45–50 Gy in 5 fractions) different from the other studies usually utilized 33.5–40 Gy in 5 fractions. Five-year BCRFS was excellent especially in 47.5 and 50 Gy arms (100%), however, ≥ grade 3 late GI and GU toxicity rates were higher than those of the other studies (Table 4). It seems that selective dose-escalation for DIL in a five-fraction setting is a feasible option to increase the biochemical control without the increment of toxicities.

**Table 4:** Summary of previous reports on the treatment results of stereotactic radiotherapy for localized prostate cancer.

Study	Year, design	N	Median age, years	Median PSA, ng/mL	Risk group	Median prostate volume, cc	Dose (Gy)/fractions	Median f/u, M	BCRFS^b^ (%)	Late toxicity (%)			
										GI/GU	G2	G3	G4
Aluwini^13^	2013, P	50	68^a^	8.2^a^	L, I	48^a^	38 and 44 (DIL)/4	23	2Y, 100 (L, I)	GI	3	0	0
										GU	10	6	0
Freeman^8^	2011, P	41	66	5.6	L	NA	35–36.25/5	60	5Y, 93	GI	2.5	0	0
										GU	7	2.5	0
Katz^9^	2014, R	515	69.5	5.4	L, I, H	59.15	35–36.25/5	72	7Y, 95.8 (L), 89.3 (I), 68.5 (H)	GI	4	0	0
										GU	9.1	1.7	0
Davis^10^	2015, R	437	69	5.8	L, I, H	52	35–38/5	20	2Y, 99 (L), 94.5 (I), 89.8 (H)	GI	2	0	0
										GU	2–8	0	0
Hannan^11^	2016, P	91	66	5.4	L, I	33	45–50/5	54	5Y, 90.9 (45 Gy), 100 (47.5–50 Gy)	GI	13.2	4.4	2.2
										GU	20.9	4.4	1.1
Kishan^12^	2019, R	2,142	68	5.7	L, I	NA	33.5–40/4–5	83	7Y, 95.5 (L), 89.8 (I)	GI	4.5	0.4	
										GU	12.3	2.4	

*BCRFS* biochemical recurrence-free survival, *DIL* dominant intraprostatic lesion, *f/u* follow-up, *G* grade, *GI* gastrointestinal, *GU* genitourinary, *H* high, *I* intermediate, *L* low, *M* months, *N* numbers, *NA* not available, *P* prospective, *PSA* prostate specific antigen, *R* retrospective, *Y* year(s).

^a^Reported as mean values.

^b^Biochemical recurrence was defined according to Phoenix definition (nadir + 2 ng/mL) in all studies.

The main hurdle for DIL boost is common invisibility in imaging. Although multiparametric magnetic resonance images (mpMRI) has an advantage of excellent tissue contrast for identifying clinically prostate cancer^21^, tumors with small-volume or low Gleason score or certain histological architectures were less likely to be detected^22^. We attributed the low number of patients with a visible tumor (27.3%) in the present study to the reasons above. However, the rapid evolvement of imaging modalities has been performed. For example, positron emission tomography and computed tomography (PET-CT) using ^11^C-labelled or ^18^F-labelled choline are increasingly being used for primary and recurrent prostate cancer^23^. Simultaneous DIL boost contoured derived by choline PET-CT has also reported its feasibility^24^. More recently, PET-CT using prostate-specific membrane antigen (PSMA) labeled with ^68^Ga was reported that had better contrast than that of choline PET-CT and could improve detection, localization of prostate cancer^25^. Go with the advances in imaging, the interest in DIL boost is expected to be increased.

The present study has a limitation of a dosimetric planning study with no clinical outcomes. However, a planning study must be preceded clinical study for the safety of the patients. Several on-going phase II studies are evaluating SIB to DIL in SBRT settings^26^, including hypo-FLAME study (NCT02853110) investigating the feasibility of 35 Gy in 5 fractions to the whole prostate with an additional boost to DIL up to 50 Gy. As the patient accrual was completed in 2018, it will take some time for maturing the data. Choosing a proper candidate is also an important issue, so we demonstrated that the location of the DIL was the main factor to do DIL boost with CK-SBRT, and to decide the dose regimen.

In conclusion, the DIL boost using CK-SBRT could be considered for localized prostate cancer patients. For patients who had DIL in posterior segments, a moderate dose escalation of 40 Gy in 5 fractions seemed appropriate. The present strategy should be validated in future clinical trials.

## Methods

### Patients

The present study was conducted in accordance with the standards and regulations of Korean Good Clinical Practice. The institutional review board of Asan Medical Center approved this study (No. 2017-0444). The requirement for informed consent to participate in the study was waived due to it retrospective design. Between July 2011 and July 2017, 55 patients were diagnosed with localized prostate cancer and received CK SBRT without a DIL boost (35 Gy/5 fractions to prostate). We selected the consecutive 15 patients who had a visible tumor on diagnostic mpMRI. Three gold fiducial markers were implanted in the prostate and 1 week after marker implantation, planning computed tomography (CT) was acquired in a thickness of 1.25 mm in a supine position. Patients were instructed to empty their bladder, and the Foley catheter was inserted for contouring urethra. Routine enema was done before planning CT scan and every SBRT session. To define the location of the DIL, we utilized 16 segments standardized MRI prostate reporting scheme from the recommendation of a European Consensus Meeting (Supplement 1)^27^.

### Contouring

For DIL delineation, T2 and diffusion-weighted MRI images were fused with the planning CT using the Varian Eclipse treatment planning system. The location of the tumor positive biopsy site was also considered. No margin was put around the DIL. Whole prostate and proximal seminal vesicles were included in clinical target volume (CTV). PTV was a 2–3 mm expansion of CTV. Bladder and rectum were delineated according to the Radiation Therapy Oncology Group consensus guideline^28^. The urethra was defined as the outer contour of the Foley catheter.

### Planning

CK plans were generated using the ray-tracing dose calculation algorithm. For the MultiPlan planning system, sequential optimization planning was performed for each patient using a 6 MV flattening-filter-free beam generated by the CK radiosurgery system (version 9.5). SIB plans of 40 Gy/5 fractions (DIL_40 plan) and 45 Gy/5 fractions (DIL_45 plan) to DIL were compared to No boost plan to the prostate (35 Gy/5 fractions). Ninety-nine percentage of PTV had to receive 80% of the prescription dose (PD) of a minimum of 28 Gy. The maximum dose must not exceed 120% of the PD (42 Gy) in the No-boost plan, 130%, and 140% of the PD for DIL_40 (45.5 Gy) and DIL_45 (49 Gy) respectively. Ninety-five percentage of the whole prostate should be irradiated ≥ 100% of the PD, and 95% of DIL volume had to receive 95% of the PD to DIL (33.25 Gy in No boost plan, 58 Gy in DIL_40 plan, 42.75 Gy in DIL_45 plan). CI was the ratio of the ‘PIV/TV_PIV_’, where the TV_PIV_ is the target volume in the prescription isodose volume (PIV). Tumor coverage (CO), defined as the ratio of the ‘TV_PIV_/TV’. We listed the constraints of the OAR in Table 1.

### Statistics

To compare 3 plans for every 15 patients, we used repeated measured ANOVA test in case normal distribution was satisfied. If not, the Friedman test was used instead. An overall p-value of < 0.05 was considered statistically significant. A significance level of multiple comparisons (p-value of < 0.05/3) was determined by Bonferroni correction. The IBM SPSS Statistics 20 package was used to perform the analysis.

## Supplementary information

Supplementary information.

## Data Availability

The datasets analyzed during the current study are available from the corresponding author on reasonable request.
