# Supplementary material for: Simultaneous integrated boost with stereotactic radiotherapy for dominant intraprostatic lesion of localized prostate cancer: a dosimetric planning study
Source: Sci Rep. 2020 Sep 7;10:14713. doi: 10.1038/s41598-020-71715-2 (PMC7477222; doi:10.1038/s41598-020-71715-2)
Supplement: Supplementary file 1 — Supplementary information. [file 41598_2020_71715_MOESM1_ESM.docx]

Supplement 1. Sixteen segments standardized magnetic resonance imaging prostate reporting scheme ^27^

**
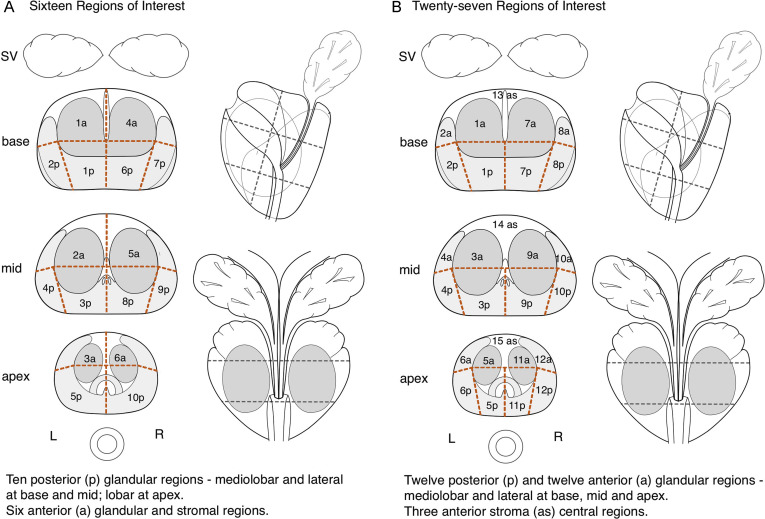
**
